# PD-L1 Expression and Comprehensive Genomic Profiling in Advanced NSCLC: A Single-Centre Experience

**DOI:** 10.3390/ijms26136348

**Published:** 2025-07-01

**Authors:** Giedrė Gurevičienė, Lina Poškienė, Skaidrius Miliauskas, Marius Žemaitis

**Affiliations:** 1 Department of Pulmonology, Medical Academy, Lithuanian University of Health Sciences, 44307 Kaunas, Lithuania; 2 Department of Pathology, Medical Academy, Lithuanian University of Health Sciences, 44307 Kaunas, Lithuania

**Keywords:** programmed death-ligand 1, tumour mutation burden, genetic profiling, STK11, KEAP1, TP53, non-small cell lung cancer, fast disease progression

## Abstract

Although immunotherapy has led to a breakthrough in the treatment of NSCLC, fast disease progression in some patients remains problematic. Great efforts are being made to identify the mechanisms of immune resistance and to establish new predictive and prognostic biomarkers. The aim of this study was to evaluate the association between PD-L1 expression, genetic alterations, and prognosis in patients diagnosed with metastatic NSCLC. PD-L1 expression and genetic profiling using NGS were assessed in 50 patients with advanced NSCLC who were negative for EGFR mutations. According to this study results, positive PD-L1 expression was detected in 62% of cases, whereas high TMB was detected in 34% of cases. Targetable mutations were detected in 33.4% of cases. The TP53 mutation was more likely to be found in tumours with higher PD-L1 and TMB levels (median 45 vs. 0, *p* = 0.005; median 10 vs. 4.5, *p* = 0.008, respectively). Meanwhile, STK11 mutation was associated with lower PD-L1 and higher TMB levels (median 0 vs. 17.5, *p* = 0.019; median 11.5 vs. 6, *p* = 0.047). Fast disease progression was observed in 22.2% of cases when immunotherapy alone or combined with chemotherapy was administered, with the most frequently detected TP53 (87.5%), STK11 (37.5%), and KEAP1 (37.5%) mutations in this part of the population. Progressive disease was more likely to be found in patients with KEAP1 mutation than in those with wild-type KEAP1 (75% vs. 18.8%, *p* = 0.02). To conclude, significant associations were found between PD-L1, TMB statuses and mutations in STK11 and TP53. Fast disease progression was established in 1/5 of the entire population treated with immunotherapy or chemo-immunotherapy. TP53, STK11, and KEAP1 mutations were most frequently detected in patients with fast disease progression. The KEAP1 mutation was associated with progressive disease in patients with advanced NSCLC. Our results suggest that a specific genetic profile could serve as a predictor of fast progression in a selected group of patients.

## 1. Introduction

Immunotherapy has led to unprecedented improvements in the survival of patients with many malignancies, including non-small cell lung cancer (NSCLC). The efficacy of immune checkpoint inhibitors (ICI) in patients with advanced NSCLC, as a single agent or in combination with chemotherapy, has been confirmed in clinical trials and is widely used in clinical practice. According to various clinical trials in advanced NSCLC, immunotherapy as monotherapy or combined with chemotherapy has significantly improved overall survival (OS) and progression-free survival (PFS), extending outcomes from several months to several years compared with chemotherapy alone [1,2]. For example, according to the results of the Impower110 study, immunotherapy in advanced NSCLC has extended OS by approximately 3–7 months, with greater benefits observed in patients exhibiting high PD-L1 expression levels [3]. Moreover, based on the statistically significant improvement in disease free survival (DFS), immunotherapy has been approved for the treatment of early-stage NSCLC [4,5]. Furthermore, perioperative immunotherapy has significantly increased the rate of pathological complete response, reaching up to 18% compared to 4.0% observed in the placebo group [6]. However, the benefits of immunotherapy in patients with advanced NSCLC remain modest, with response rates (RR) of 30–45%, 5-year survival rate of 13–32%, depending on programmed death ligand one (PD-L1) levels, when immunotherapy is administered as a single agent [7,8]. Additionally, when immunotherapy is administered in combination with chemotherapy, the RR reaches 20–47%, depending on the PD-L1 level, but 5-year survival remains only up to 20% [8,9]. PD-L1 is the only biomarker currently approved for clinical use to select patients for immunotherapy in the monotherapy setting [10]. However, the reliability of PD-L1 expression as a single biomarker for clinical decision-making in patients with NSCLC is insufficient [11].

Recent studies have comprehensively examined tumour molecular profiling, emphasising the tumour mutation burden (TMB), which is the number of somatic non-synonymous mutations found in the DNA of cancer cells, expressed in mutations per megabase (muts/Mb). TMB has shown great potential as a predictive biomarker for the efficacy of ICI in NSCLC [12]. A higher TMB is considered to play a much greater role in the genesis of tumour neoantigens, allowing heightened recognition of malignant cells as “non-self” and a more effective anti-tumour immune response. TMB has been consistently analysed as a potential additional biomarker for treatment. However, several studies and recent updates in clinical guidelines have advised against the use of TMB as a predictive biomarker due to its inconsistent correlation with therapeutic response and survival. Consequently, the reliability and feasibility of TMB in this context remain uncertain [11,13,14,15].

Further research has demonstrated that certain genetic alterations can also serve as predictive biomarkers. Two types of genes can be distinguished in terms of cancer development: oncogenes and tumour suppressor genes [16]. The development of targeted therapies against driver oncogene mutations has played a valuable role in genetic mutation research. These findings underscore the relevance of non-oncogene-driven mutations in the molecular landscape of NSCLC. The two most common non-oncogene-driven mutations in lung adenocarcinoma, STK11 (serine/threonine kinase 11) and KEAP1 (Kelch-like ECH-associated protein 1) mutations, were found to be predictors of poor outcomes in patients with NSCLC treated with immunotherapy [17]. However, further studies have revealed that STK11 and KEAP1 mutations are associated with lower survival rates regardless of treatment; therefore, the results remain controversial [18]. Moreover, not only single mutations but also combinations of these mutations were crucial in the patient’s response to immunotherapy. A significantly lower survival rate was found in NSCLC with STK11 and KEAP1 mutations among KRAS (Kirsten rat sarcoma virus)-mutated, but not KRAS wild-type patients, treated with immunotherapy [19]. In addition, survival in patients with KRAS-mutated tumours also depends on PD-L1 levels, with favourable results for those with high PD-L1 expression [20]. Furthermore, patients with high TMB and at least one STK11/KEAP1/EGFR (epidermal growth factor receptor) alteration demonstrated durable treatment benefits [21]. Therefore, while the selection of patients for immunotherapy remains challenging, ongoing research evaluating the efficacy of combined biomarkers is crucial for improving our understanding of cancer biology and developing new therapeutic strategies for NSCLC treatment.

The absence of treatment response and the occurrence of fast disease progression within the first three months of initiating immunotherapy in NSCLC patients remain clinically significant challenges. Fast progression is likely related to primary tumour resistance to immunotherapy, underscoring the importance of ongoing research aimed at identifying reliable predictive biomarkers [22,23,24].

The aim of this study was to evaluate the association between PD-L1 expression, genetic alterations, and prognosis in patients with metastatic NSCLC.

## 2. Results

PD-L1 expression, TMB, and gene alterations were assessed in 50 tumour samples from patients with metastatic NSCLC, obtained at the time of diagnosis, prior to therapy, previously tested according to a standard clinical protocol by PCR, and negative for EGFR mutation. The baseline characteristics of the patients are shown in Table 1. The median follow-up time of the entire cohort was 17 months (range, 12.9–20.1 months).

In most cases, the biopsy samples were taken from a primary lung lesion; intrapulmonary lymph nodes or distant metastases were used in less than one-third of cases. Immunotherapy, either as a single agent or in combination with chemotherapy, was administered in 72% of cases. Targeted therapy was administered to patients whose tumours harboured ALK or EGFR mutations, in accordance with the local reimbursement policy, in 6% of cases. Positive PD-L1 expression was detected in two-thirds of the cases (n = 31, 62%). PD-L1 levels were <1%, 1–49%, and ≥50% in 19 (38%), 13 (26%), and 18 (36%) patients, respectively. The median TMB was 7 muts/MB. High TMB was detected in 1/3 of the cases (n = 17, 33.3%). The results of TMB are presented in Figure S1.

**Figure 1:**
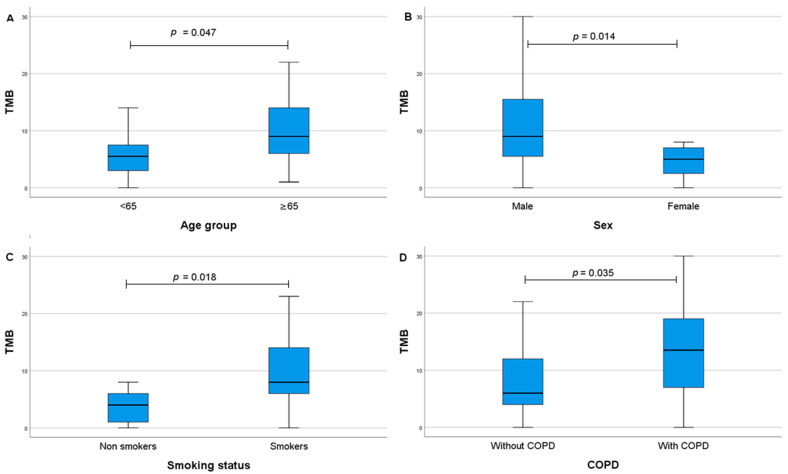
The association between TMB and clinicopathological features: age group (**A**), sex (**B**), smoking status (**C**), and COPD (**D**). TMB—tumour mutation burden, COPD—chronic obstructive pulmonary disease.

Significantly higher TMB levels were found in patients in the age group of ≥65 years compared to younger patients (median 9 vs. 6, *p* = 0.047); men compared to women (median 9 vs. 5, *p* = 0.014); smokers compared to non-smokers (median 8 vs. 4, *p* = 0.018); and patients diagnosed with chronic obstructive pulmonary disease (COPD) compared to patients without COPD (median 13.5 vs. 6, *p* = 0.035) (Figure 1A–D). Similar associations were observed when comparing high or low TMB levels and clinicopathological characteristics: high TMB levels were more likely to be found in men and patients ≥65 years of age (Table S1).

Moreover, a significant correlation was found between TMB and pack-years (R = 0.542, *p* = 0.001). A higher number of pack-years was more likely to be found in patients with high TMB than in those with low TMB (median 40 vs. 15, *p* = 0.021) (Figure S2). The median TMB in tumours with negative PD-L1 expression was 6 mut/Mb, and 8 mut/Mb for patients with positive PD-L1 expression. No significant correlations or associations were found between PD-L1 expression and TMB.

**Figure 2:**
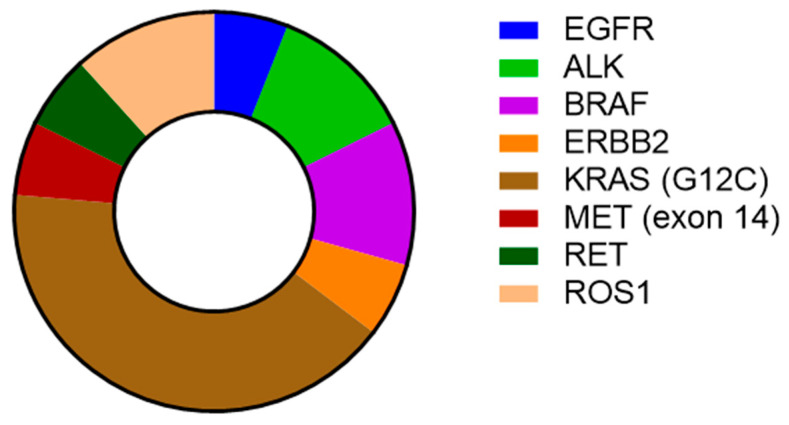
Activating mutations presented as a percentage.

A total number of 311 alterations in 111 genes were detected in 50 patients. Only one case showed no alterations (n = 1, 2%). Targetable mutations were observed in 33.4% of cases: EGFR (n = 1, 2%), ALK (anaplastic lymphoma kinase) (n = 2, 3.9%), BRAF (n = 2, 3.9%), (n = 1, 2%), KRAS G12C (n = 7, 13.7%), MET (exon 14) (mesenchymal epithelial transition factor) (n = 1, 2%), RET (rearranged during transfection) (n = 1, 2%), and ROS1 (c-ros oncogene 1) (n = 2, 3.9%) (Figure 2). All activating mutations were detected only in the adenocarcinoma histological NSCLC type.

**Figure 3:**
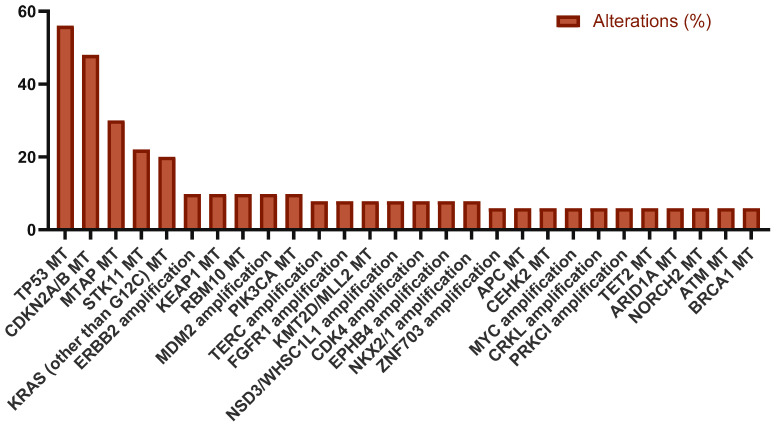
Other most common mutations in metastatic NSCLC.

The frequency of TP53 (transformation-related protein 53) mutation was the highest (n = 28, 56%), followed by CDKN2A/B (cyclin-dependent kinase inhibitor 2A/B) (n = 24, 48%), MTAP (S-methyl-5′-thioadenosine phosphorylase) (n = 15, 30%), KRAS (other than G12C) (n = 10, 20%), and STK11 (serine/threonine kinase 11) (n = 11, 22%) (Figure 3).

MT—mutation.

The probability of detecting TP53 mutations was significantly higher in the adenocarcinoma NSCLC histological type, with similar tendencies for STK11 and KRAS mutations. Meanwhile, TERC (telomerase RNA component), FGFR1 (fibroblast growth factor receptor 1), and NSD3/WHSC1L1 (nuclear receptor binding SET domain protein 3/Wolf-Hirschhorn syndrome candidate 1-like 1) amplifications, as well as KMT2D/MLL2 (Histone-lysine N-methyltransferase 2D/Mixed lineage leukaemia 2) mutations, were more likely to be found in squamous cell carcinomas (Table 2).

Co-occurring mutations in KRAS/STK11, KRAS/TP53, STK11/KEAP1, and STK11/TP53 were found in 10% (n = 5), 8% (n = 4), 6% (n = 3), and 14% (n = 7) of cases, respectively. KRAS/STK11 and STK11/KEAP1 mutations were more likely to be found in NSCLC without PD-L1 expression (Table 3).

**Figure 4:**
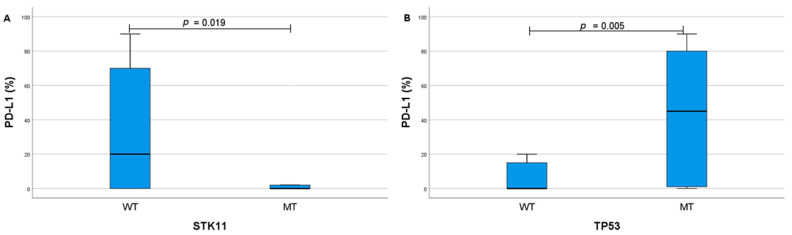
Associations between PD-L1 expression and mutations in STK11 (**A**), and TP53 (**B**). PD-L1—programmed death ligand 1, WT—wild type, MT—mutation.

**Figure 5:**
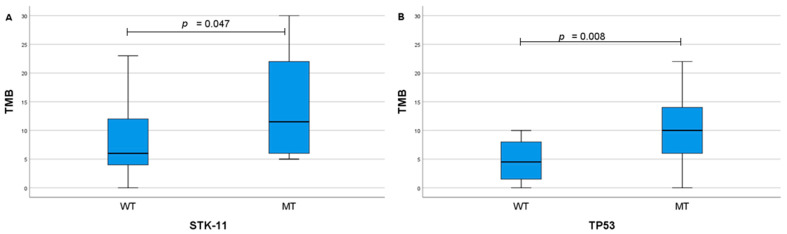
Associations between TMB and mutations: STK11 (**A**), and TP53 (**B**). TMB—tumour mutation burden, WT—wild type, MT—mutation.

Patients with lower PD-L1 expression levels were more likely to have STK11 mutations (median 0 vs. median 17.5, *p* = 0.019) (Figure 4A). Meanwhile, the TP53 mutation was more likely to be found in patients with higher PD-L1 levels (median 45 vs. 0, *p* = 0.005) (Figure 4B). Moreover, both STK11 and TP53 mutations were more common in patients with higher TMB levels (median 11.5 vs. 6, *p* = 0.047; median 10 vs. 4.5, *p* = 0.008, respectively) (Figure 5A,B). Similar tendencies were observed when evaluating the associations between PD-L1 expression groups (positive or negative; low or high) and genetic alterations (Table 4).

Notably, only cases in which immunotherapy or chemo-immunotherapy was administered were included in the evaluation of the impact of PD-L1, TMB, and genetic alterations alone or in combination on patients’ RR, PFS, and OS. Accordingly, 36 patients met the inclusion criteria. Assessment of these 36 patients showed a partial response (PR) in 27.8% (n = 10) of cases, stable disease (SD) in 47.2% (n = 17) of cases, and progressive disease (PD) in 25% (n = 9) of cases. Considering PD-L1 expression, PR was more frequently observed in patients with positive PD-L1 expression than in those with negative PD-L1 expression (41% versus 7%); however, the difference was not statistically significant (Table S2).

Progressive disease, classified as fast progression within three months from the beginning of treatment, was detected in eight patients. The characteristics of these patients are represented in Table 5. It is important to note that, in addition to the most common TP53 mutation, these cases were linked to STK11 or KEAP1 mutations in 62.5% of patients. Among patients with both STK11 and KEAP1 wild-type tumours, a low TMB and alterations in the ERBB2 pathway were detected in 66.7% of cases.

While previous studies have demonstrated that fast disease progression could be associated with gene alterations, leading to tumour microenvironment changes and altered immune response, the study population was grouped into fast progression and non-fast progression subgroups. The results of these subgroup analyses, including the most frequently detected mutations and those reported to be associated with fast disease progression, are presented in Table 6. Fast disease progression was significantly more likely to be found in patients with KEAP1 mutation than in those with KEAP1 wild type (75% vs. 15.6%, *p* = 0.028). The same trend, although insignificant, was observed for ERBB2 amplification (Table 6).

While previous studies have demonstrated that co-occurring mutations could also serve as predictive biomarkers, patients were divided into subgroups according to the most common co-occurring mutations. However, no significant association was found between co-occurring mutations and response to therapy (Table S3).

Further analysis revealed that PD-L1 expression, TMB, and genetic alterations alone or in combination were not associated with PFS or OS.

## 3. Discussion

This study aimed to evaluate the association between PD-L1 expression, genetic alterations, and prognosis in patients with metastatic NSCLC.

In advanced NSCLC, positive PD-L1 expression, evaluated by TPS, usually distributes as one-third in the PD-L1 <1% group, one-third in the PD-L1 1% to 49% group, and one-third in the PD-L1 ≥50% group [25]. This is in line with our results of PD-L1 expression, when PD-L1 <1%, 1–49%, and ≥50% were detected in 38%, 26%, and 36% of cases, respectively. According to our study results, no association between TMB and PD-L1 expression was found, which corresponds to the majority of previous studies [26].

The median TMB that we detected in patients with metastatic NSCLC was 7 muts/Mb, which is similar to the results reported in another real-world clinical study of 667 patients with metastatic NSCLC, where the median TMB was 8 muts/Mb [27]. The results of clinical trials evaluating TMB as a predictive biomarker suitable for immunotherapy selection remain controversial. The FDA has approved pembrolizumab for the treatment of unresectable or metastatic solid tumours with high TMB that have progressed after prior treatment and have no alternative therapy options [28]. However, a series of further studies revealed that TMB is not a reliable biomarker in patients with advanced NSCLC, which was confirmed by the results of our research. The CheckMate 227 clinical trial results question the role of TMB as a predictive biomarker for treatment selection in advanced NSCLC, as there was no significant association between high TMB and OS [29]. Data discrepancies could occur due to the use of different panels for TMB testing and different cut-off values defining low and high TMB. For example, we used the FoundationOne CDx panel, which includes 324 genes and is confirmed for TMB testing in various types of tumours, including NSCLC. Meanwhile, the Memorial Sloan Kettering-Integrated Mutation Profiling of Actionable Cancer Targets (MSK-IMPACT) panel includes a broader set of genes and can also be used for TMB testing. In the FoundationOne CDx assay, ≥10 muts/Mb indicates a high TMB, as well as in clinical trials evaluating pembrolizumab as a first-line therapy for NSCLC. However, in a clinical trial evaluating nivolumab combined with ipilimumab in the same setting, a high TMB was defined as ≥13 mut/Mb [11,28]. To use TBM in daily clinical practice, an unambiguous definition of TMB calculation and harmonisation of the different TMB assay methods are required.

Assessing targetable mutations, KRAS G12C was the most common oncogenic driver mutation, with an incidence of 13.7% (41.2% of all KRAS mutations), while other than G12C mutations were detected in 20% of cases. This is in line with the results of other studies, where the KRAS mutation was found in up to 30% of cases, with an incidence of KRAS G12C in up to 40% of all KRAS mutations [30]. Evaluating non-targetable mutations, the most common was the TP53 mutation (56%), followed by CDKN2A/B (48%), MTAP (30%), KRAS (other than G12C) (20%), and STK11 (22%). This result is consistent with that of previous studies, where TP53 is known to be one of the most frequent mutations detected in NSCLC, with an incidence of up to 55% [31,32], followed by STK11 with an incidence of 25–30% [33]. Interestingly, while in advanced NSCLC, MTAP loss and CDKN2A/B alteration are reported in up to 13% and 19% of cases [34,35], we found a frequency of 30% and 48%, respectively. While studies representing a lower rate of these mutations mostly contain adenocarcinoma histological type, the higher numbers of MTAP and CDKN2A/B alteration incidences in our case could be due to squamous NSCLC patients, who accounted for 1/3 of all patients.

Evaluating co-occurring mutations depending on PD-L1 status, in PD-L1 negative tumours, KRAS/STK11 mutation was detected in 10% and STK11/KEAP1 mutation in 6% of cases, which was consistent with previous clinical study results. These findings also highlighted that KRAS/STK11 and STK11/KEAP1 co-occurring mutations were more likely to be found in tumours with negative PD-L1 expression [36].

The association between KRAS/STK11 mutations and PD-L1 expression is explained through pro-inflammatory cytokine (IL6, CXCL-7, and G-CSF) overproduction, which leads to an increased number of neutrophils with T-cell suppressive function, resulting in lower IFNγ secretion, which induces PD-L1 expression. Meanwhile, the KEAP1 mutation alone is reported to be associated with tumours expressing higher PD-L1 levels. The CUL3–KEAP1 complex interferes with the ubiquitination of nuclear factor erythroid 2-related factor 2 (NRF2), leading to increased PD-L1 transcript expression [37]. In contrast, co-occurring STK11/KEAP1 mutations, probably due to the STK11 impact on “cold” tumour microenvironment, lead to lower levels of PD-L1 expression and decreased response to immunotherapy.

The majority of studies have demonstrated a favourable response and better outcomes for patients treated with immunotherapy for tumours expressing higher PD-L1 levels. Our study results also demonstrated a trend of better RR in patients with positive PD-L1 expression. Statistical significance was not reached probably due to a small sample size. However, other large-scale clinical trials have also demonstrated that patients with negative PD-L1 expression still benefit from immunotherapy alone or in combination with chemotherapy [29]. This could be due to the non-canonical PD-1/PD-L1 pathway, which is represented by PD-L1 expression on T cells and PD-1 expression on tumour and myeloid cells, which exhibit extraordinary protein interactions, signaling pathways, and cell crosstalk, resulting in the regulation of cell growth, differentiation, and metabolism [38].

In our study evaluating the best overall response rate, disease progression was observed in 25% of cases, and 22.2% of cases from the entire cohort treated with immunotherapy alone or combined with chemotherapy met the criteria for fast disease progression. Based on previous clinical trial results, the incidence of fast disease progression varies from 10% to 42% [22,24,29]. This could be due to primary tumour resistance to therapy. According to our data, five of the eight patients who experienced fast disease progression were detected with STK11 and/or KEAP1 mutations. Moreover, the TP53 mutation was found in seven of eight cases, and in four cases, they were accompanied by STK11 and/or KEAP1 mutations. In contrast, among patients with both STK11 and KEAP1 wild-type tumours, low TMB and alterations in the ERBB2 pathway were detected in 2/3 of cases.

Our study results revealed that patients with KEAP1 mutations were more likely to be diagnosed with PD than those without. The KEAP1 mutation is reported to be associated with worse prognosis, shorter DFS, and OS [39]. KEAP1 regulates the NRF2L2 (nuclear factor (erythroid-derived 2)-like 2) pathway, which plays a crucial role in the antioxidant defense mechanism. A murine model of lung carcinoma cell lines demonstrated that KEAP1 deletion in tumours with inactivated TP53 is associated with chemoresistance through the KEAP1-NFE2L2 pathway. Mutations in KEAP1 lead to uncontrolled activation of NRFE2L2, which targets genes involved in xenobiotic biotransformation reactions and antioxidant metabolism, thereby protecting tumour cells from the effects of cytotoxic chemotherapy [40]. Moreover, comprehensive research evaluating 450 cases of lung adenocarcinoma with KEAP1 mutations revealed that KEAP1-mutated tumours were associated with lower tumour-infiltrating lymphocytes and cytotoxic T lymphocyte infiltration in tumour tissue. Moreover, KEAP1 mutation was associated with lower human leukocyte antigen (HLA) expression and lower enrichment scores of 18 immune-related pathways, including the IL-17 signaling pathway, complement and coagulation cascade, platelet activation, toll-like receptor signalling and other pathways, resulting in a cold tumour microenvironment, with a lower likelihood of responding to immunotherapy.

The underlying mechanism of STK11 mutation and fast disease progression may be related to the association of STK11 with PD-L1 and TMB. According to our data, the STK11 mutation was more likely to be found in tumours expressing lower levels of PD-L1 and higher levels of TMB. This is consistent with previously published scientific data. Lamberti et al. in a study of NSCLC, which included 909 patients with most non-squamous histology and advanced stage cases, demonstrated that tumours with negative PD-L1 expression, compared to those with high PD-L1 expression, were associated with mutations in STK11 [41]. In the KEYNOTE-189 study, in which 18.7% of 289 evaluable patients had the STK11 mutation, PD-L1 levels evaluated by TPS tended to be lower and TMB tended to be higher in patients with STK11 mutations than in those without [42].

The STK11 gene, also known as LKB1, encodes a protein serine/threonine kinase that behaves as a tumour suppressor and is also responsible for multiple biological and cellular processes, including metabolism maintenance, cell polarity, proliferation, and migration. Loss of STK11 function due to mutation is common in many tumour types, including NSCLC, with an incidence of up to 15% of patients [19,39]. STK11 mutations are mostly enriched in tumours, expressing high TMB levels with a “cold” tumour immune microenvironment, characterised by reduced density of effector T lymphocytes and increased density of neutrophils [19,43,44]. A cold tumour microenvironment is the most common explanation for the association between STK11 mutation and low PD-L1 expression, as well as a worse response to immunotherapy. In a KRAS-driven murine model of NSCLC and human cell lines, STK11-mutated tumours overproduced IL6, CXCL-7, and G-CSF, known as pro-inflammatory cytokines, which determine the accumulation of neutrophils with T-cell suppressive function [44,45]. In colorectal cancer, where a murine isograft model was used, STK11 modulated TGFβ signalling, which mediated the exclusion of T-cells and suppressed the differentiation of Th1 cells, resulting in a “cold” tumour immune microenvironment [43]. Deng et al. revealed in vitro experiments that STK11 loss induced DNA double-strand break repair defects via homologous recombination. STK11 mutated tumours facilitate immune evasion by reducing antigen presentation through decreasing the expression of immunoproteasome components that generate immunogenic peptides for presentation by MHC-1 [46]. At this point, lower PD-L1 expression can be indirectly linked to STK11 mutation due to lower T-cell activity and smaller amount of IFNγ secreted by these cells, which is known as a cytokine, inducing PD-L1 expression in STK11 mutated tumours [44].

The association between STK11 mutation and high TMB can occur due to damaged DNA repair mechanisms through the mechanistic target of rapamycin (mTOR) and AMP-activated protein kinase (AMPK) pathways [47].

A series of clinical trials have evaluated the role of STK11 and KEAP1 mutations as predictive or prognostic biomarkers; however, the results remain controversial. Evaluating the impact of STK11 and KEAP1 mutations on the outcomes of ICI and other therapies, the results showed a similar effect regardless of the treatment received, suggesting prognostic, rather than predictive, value [33].

Moreover, clinical data indicate that despite the fact that STK11-mutated tumours usually exhibit high TMB levels, which are considered to be associated with a favourable response to immunotherapy, these tumours have worse response rates to immunotherapy and are associated with poor prognosis in NSCLC [46]. Other clinical studies suggest that the poor prognosis of patients with STK11 mutations is primarily linked to co-occurring KRAS mutations, but not STK11 mutations alone [33]. However, the co-occurring KRAS/STK11 mutation for patients with fast disease progression in our study was not detected. These controversial results could also be related to the fact that in a subset of lung adenocarcinomas, a pathway of non-mutational STK11 inactivating mechanisms also exists [48]. Interestingly, the mutation type and location can be associated with different prognoses; STK11 mutations in exons 1 and 2 are associated with an inferior prognosis compared to mutations occurring in exons 3–9 [39].

Meanwhile, TP53 mutation, especially co-mutation with STK11 or KEAP1, is reported to be associated with worse prognosis [49]. The mechanism of TP53 mutation is also related to PD-L1 expression and TMB. Tumours with TP53 mutations are associated with increased TMB and higher levels of PD-L1 expression, which was also validated in our research. TP53 encodes the p53 tumour suppressor protein, which plays a crucial role in the cell cycle and maintenance of genomic stability [32]. In a study where data of 563 NSCLC patients’ obtained from The Cancer Genome Atlas were analysed, only patients with TP-53 mutation, regardless of their type, were associated with higher TMB [50]. In contrast, the same study demonstrated that only TP53-missense-mutation, but not TP53 nonsense mutations, had increased IFN-γ levels, followed by increased PD-L1 levels, compared with the subgroup without TP53 mutation [50]. These findings lead to the assumption that different TP53 mutations shape different tumour microenvironments, which may lead to different responses to immunotherapy. Interestingly, in a study where 340 cases of NSCLC in the Chinese population were evaluated, higher TMB levels were observed in tumours without TP53 mutations but not in TP53-mutated tumours [51]. However, another study in the Chinese population, which included 469 lung adenocarcinoma cases, demonstrated significantly higher TMB levels in TP53-mutated tumours [52].

The association between higher PD-L1 expression levels in TP53 mutated tumours may be due to the fact that TP53 encodes a protein that increases miR-34 expression, an miRNA involved in the attenuation of PD-L1 expression through its binding to the 3′ untranslated regions of PD-L1 mRNA in models of NSCLC [44,53]. In an isogenic melanoma cell model TP53 mutation resulted in higher PD-L1 expression levels through the SOX10/IRF1 regulatory axis [54].

Meanwhile, the association between TP53 and TMB may be due to previously reported TP53 gene mutation involvement in cell growth, apoptosis, and dysfunction of DNA repair, resulting in higher TMB levels.

ERBB2, also known as HER2, is an activating oncogenic mutation that causes enhanced activation of the HER2 tyrosine kinase receptor, leading to ligand-independent signalling through the MAPK (mitogen-activated protein kinase) and PI3K/AKT (phosphatidylinositol-3 kinase/AKT) pathways. ERBB2 amplification refers to an increased number of ERBB gene copies, leading to overexpression of the HER2 protein and enhanced downstream signalling activity [55]. We found ERBB2 mutations in 2% of cases, amplifications in 8% of cases, and co-occurring mutations and amplifications in 2% of cases in the entire cohort. This is in line with other clinical studies that demonstrated similar numbers of the ERBB2 mutation, alteration, and co-occurring mutations and alterations in NSCLC patients, with incidences of 1 to 4%, from 2 to 10% and up to 5.5%, respectively [55,56]. It is important to note that analysing fast disease progression cases in our research, both patients with STK11 and KEAP1 wild-type tumours had low TMB and harboured ERBB2 alterations (amplification and co-occurring alteration). According to the literature, ERBB2 is associated with younger age, female sex, lower TMB, and PD-L1 levels, which is linked to a non-smoking history, leading to reduced exposure to mutagens, such as tobacco smoke [55]. However, when comparing tumours with ERBB2 mutations and ERBB2 amplification, higher TMB levels were observed in tumours with ERBB2 amplification. Moreover, a tendency for higher CD4^+^ T cells and CD8^+^ T cells in tumours harbouring ERBB2 amplification was detected. These findings suggest that the mechanisms of action of these genetic factors may differ, leading to different responses to treatment. Meanwhile, clinical studies evaluating treatment outcomes in NSCLC patients with ERBB2 alterations are lacking evidence; therefore, the results are controversial [57]. Our findings also suggest that ERBB2 alterations can be associated with fast disease progression due to lower TMB levels, leading to a worse response to immunotherapy.

The main limitation of our study was the small sample size and relatively short follow-up period. Due to these limiting factors, significant differences between prognosis and gene alterations, as well as their combination with PD-L1 expression, could not be reached. However, we believe that the associations found between PD-L1 expression, TMB, and genetic alterations, as well as genetic alterations associated with response rate and specific genomic profiles in fast disease progression, provide reasonable evidence to justify further research with a larger sample size, continuing the search for reliable prognostic biomarkers to facilitate the selection of NSCLC treatment solutions.

## 4. Materials and Methods

### 4.1. Methods

Ethical approval for this research protocol was obtained from the Kaunas Regional Ethics Committee for Biomedical Research (No. BE-2-44). All patients signed an informed consent form for their tumour samples and clinical data to be collected in this study.

### 4.2. Study Population

Tumour tissue specimens from 50 patients diagnosed with stage IV NSCLC at the Hospital of Lithuanian University of Health Sciences, Kaunas Clinics, between January 2023 and September 2024 were included in the study. Tumour tissue specimens were collected at the time of NSCLC before treatment and tested for EGFR according to a standard clinical protocol by PCR, and patients with an EGFR mutation were not included in the study.

The classification of tumours stage was based on the 8th edition of the TNM Classification of Malignant Tumours [58]. The World Health Organization (WHO) classification of lung tumours was used to classify lung tumour histology [59]. Patients’ clinical and pathological information was obtained from medical documentation (clinical records and pathology reports).

Smoking status was determined based on the number of cigarettes smoked during their lifetime. Those participants who had smoked ≥100 cigarettes in their lifetime were classified as smokers (either present or past); those who had not were classified as non-smokers. For quantification of smoking intensity, pack-years were used: the number of packs of cigarettes the participant smoked each day multiplied by the number of years.

### 4.3. Immunohistochemistry Analysis

Blocks for immunohistochemistry (IHC) staining were obtained from the lungs, mediastinal lymph nodes, or distant metastases. Formalin-fixed, paraffin-embedded (FFPE) tissue from NSCLC biopsies was sectioned into slices of 3–5 µm thickness. PD-L1 IHC analysis was performed on the Roche Ventana Benchmark XT automated slide stainer (Ventana Medical Systems, Roche, Neuilly-sur-Seine, France) using murine monoclonal anti–PD-L1 antibody, clone 22C3 pharmDx (Agilent Technologies/Dako, Carpinteria, CA, USA), according to the manufacturer’s instructions. Positive controls of human tonsils and placenta were used for each slide. PD-L1 IHC was evaluated using a light microscope (Olympus BX50 microscope (Olympus Co., Tokyo, Japan)). PD-L1 expression was assessed using the tumour proportion score (TPS), which is the percentage of viable tumour cells with any intensity of partial linear or complete circumferential expression of PD-L1 relative to all viable tumour cells within the analysed section.

### 4.4. Targeted Tumour Next-Generation Sequencing

Gene expression profiling and TMB were assessed in unstained formalin-fixed paraffin-embedded NSCLC specimens using the FoundationOne CDx assay (Foundation Medicine, Cambridge, MA, USA). Tumours with TMB ≥ 10 mutations/megabase (mut/Mb) were considered TMB high, based on previous research results [60]. 

### 4.5. Clinical Outcomes

Clinical endpoints included RR, PFS, and OS. Tumour response was assessed according to the RECIST 1.1 criteria [61]. PFS was defined as the period from the beginning of therapy to disease progression or death. Patients without disease progression at the time of data analysis were censored at the date of the last disease assessment, showing no radiological or clinical progression. OS was defined as the time period from the beginning of therapy until death. For patients who were still alive at the time of record evaluation, censoring was performed on the date of the last contact. Patients with activating mutations, including those who received targeted therapy and those who did not receive specific treatment, were excluded from the response rate and survival analyses. Fast disease progression was determined as an increase in the largest tumour diameter ≥50% within 6 weeks from the beginning of treatment (follow-up radiological assessments were performed over time to rule out pseudoprogression) or death due to cancer progression within 12 weeks, including cases when post-baseline computed tomography was absent, in accordance with previously published methodology [22].

### 4.6. Statistical Analysis

Statistical Package for the Social Sciences (SPSS) version 25 was used to perform statistical analyses. Sample normality was assessed using the Kolmogorov–Smirnov test. The median with minimum and maximum values for non-normally distributed data was used. Mann–Whithey U test was used to evaluate the differences between two independent variables. In cases of more than two independent variables, differences were assessed using the Kruskal–Wallis test. The chi–square (χ^2^) test and Fisher’s exact test were used for categorical data analysis. The Spearman rank test was used to assess the correlation between continuous variables. The survival rate was estimated using the Kaplan–Meier method. Multivariate survival analysis was performed using the Cox proportional hazards model. A *p*-value of <0.05 was considered statistically significant.

## 5. Conclusions

Positive PD-L1 expression was detected in 2/3 of the cases, and high TMB was detected in 1/3 of the cases. Targetable mutations were detected in 1/3 of the cases. STK11 and TP53 mutations were significantly associated with PD-L1 expression and TMB in metastatic NSCLC patients. Fast disease progression was observed in 22.2% of the population treated with immunotherapy alone or in combination with chemotherapy. The most frequent mutations found in this population were in TP53, followed by STK11 and KEAP1. The KEAP1 mutation was associated with fast progression in advanced NSCLC. Our findings suggest that a specific genetic profile could serve as a predictor of fast progression in a selected group of patients. Further research is required to evaluate these biomarkers as predictive or prognostic markers.

## Figures and Tables

**Table 1:** Baseline characteristics of the study population.

Baseline Characteristics	*n* (%)
Sex	
Male	38 (76)
Female	12 (24)
Age group	
<65 years	23 (46)
≥65 years	27 (54)
Smoking status	
Non-smokers	10 (20)
Current or former smokers	40 (80)
COPD	
Absent	39 (78)
Present	11 (22)
Histological NSCLC type	
Adenocarcinoma	38 (76)
Squamous cell carcinoma	10 (20)
Large cell carcinoma	2 (4)
Differentiation	
Well-moderate	22 (44)
Poor-undifferentiated	18 (36)
NSCLC stage	
IVa	21 (42)
IVb	29 (58)
T status	
T1	8 (16)
T2	7 (14)
T3	11 (22)
T4	24 (48)
Lymph node status	
N0	4 (8)
N1	7 (14)
N2	14 (28)
N3	25 (50)
Metastases	
M1a	15 (30)
M1b	6 (12)
M1c	29 (58)
Biopsy localisation	
Lung	36 (72)
Metastasis	14 (28)
Treatment	
Immunotherapy	11 (22)
Chemotherapy + immunotherapy	25 (50)
Targeted therapy	3 (6)
Not given	11 (22)

COPD—chronic obstructive pulmonary disease.

**Table 2:** Associations between genetic alterations and histology.

Alteration	Histology		*p*
	Adenocarcinoma	Squamous Cell Carcinoma	
EGFR			
WT	35 (77.8)	10 (22.2)	1
MT	1 (100)	0 (0)	
Amplification	2 (100)	0 (0)	
ALK			
WT	36 (78.3)	10 (21.7)	1
MT	2 (100)	0 (0)	
BRAF			
WT	36 (78.3)	10 (21.7)	1
MT	2 (100)	0 (0)	
ERBB2			
WT	33 (76.7)	10 (23.3)	0.655
MT	1 (100)	0 (0)	
Amplification	4 (100)	0 (0)	
KRAS			
WT	22 (73.3)	8 (26.7)	0.097
MT	15 (93.8)	1 (6.3)	
Amplification	1 (50)	1 (50)	
MET			
WT	36 (78.3)	10 (21.7)	1
MT	1 (100)	0 (0)	
Amplification	1 (100)	0 (0)	
RET			
WT	37 (78.7)	10 (21.3)	1
MT	1 (100)	0 (0)	
ROS1			
WT	36 (78.3)	10 (21.7)	1
MT	2 (100)	0 (0)	
STK11			
WT	28 (73.7)	10 (26.3)	0.094
MT	10 (100)	0 (0)	
TP53			
WT	21 (100)	0 (0)	0.003
MT	17 (63)	10 (37)	
ZNF703			
WT	36 (80)	9 (20)	0.512
Amplification	2 (66.7)	1 (33.3)	
TERC			
WT	37 (84.1)	7 (15.9)	0.025
Amplification	1 (25)	3 (75)	
MTAP			
WT	25 (75.8)	8 (24.2)	0.472
MT	13 (89.7)	2 (13.3)	
CDKN2A/B			
WT	19 (79.2)	5 (20.8)	1
MT	19 (79.2)	5 (20.8)	
FGFR1			
WT	37 (84.1)	7 (15.9)	0.025
Amplification	1 (25)	3 (75)	
KEAP1			
WT	35 (79.5)	9 (20.5)	1
MT	3 (75)	1 (25)	
KMT2D/MLL2			
WT	37 (84.1)	7 (15.9)	0.025
MT	1 (25)	3 (75)	
NSD3/WHSC1L1			
WT	37 (84.1)	7 (15.9)	0.025
Amplification	1 (25)	3 (75)	
RBM10			
WT	33 (76.7)	10 (23.3)	0.569
MT	5 (100)	0 (0)	
CDK4			
WT	36 (80)	9 (20)	0.512
Amplification	2 (66.7)	1 (33.3)	
MDM2			
WT	33 (76.7)	10 (23.3)	0.569
Amplification	5 (100)	0 (0)	
PIK3CA			
WT	34 (81)	8 (19)	0.254
MT	4 (80)	1 (20)	
EPHB4			
WT	35 (77.8)	10 (22.2)	1
Amplification	3 (100)	0 (0)	
NKX2/1			
WT	34 (77.3)	10 (22.7)	0.566
Amplification	4 (100)	0 (0)	
APC			
WT	35 (77.8)	10 (22.2)	1
MT	3 (100)	0 (0)	
CEHK2			
WT	35 (77.8)	10 (22.2)	1
MT	3 (100)	0 (0)	
MYC			
WT	35 (77.8)	10 (22.2)	1
Amplification	3 (100)	0 (0)	
CRKL			
WT	37 (82.2)	8 (17.8)	0.106
Amplification	1 (33.3)	2 (66.7)	
PRKCI			
WT	37 (82.2)	8 (17.8)	0.106
Amplification	1 (33.3)	2 (66.7)	
TET2			
WT	36 (80)	9 (20)	0.512
MT	2 (66.7)	1 (33.3)	
ARID1A			
WT	35 (77.8)	10 (22.2)	1
MT	3 (100)	0 (0)	
NORCH2			
WT	35 (77.8)	10 (22.2)	1
MT	3 (100)	0 (0)	
ATM			
WT	36 (80)	9 (20)	0.512
MT	2 (66.7)	1 (33.3)	
BRCA1			
WT	35 (77.8)	10 (22.2)	1
MT	3 (100)	0 (0)	

**Table 3:** Associations between PD-L1 expression and co-occurring mutations.

Co-Occurring Mutations	PD-L1			PD-L1		
	Negative	Positive	*p*	Low	High	*p*
KRAS/STK11						
Not detected	14 (31.1)	31 (68.9)	0.005	27 (60)	18 (40)	0.148
Detected	5 (100)	0 (0)		5 (100)	0 (0)	
KRAS/TP53						
Not detected	17 (37)	29 (63)	0.629	30 (65.2)	16 (34.8)	0.612
Detected	2 (50)	2 (50)		2 (50)	2 (50)	
STK11/KEAP1						
Not detected	16 (34)	31 (66)	0.049	29 (61.7)	18 (38.3)	0.544
Detected	3 (100)	0 (0)		3 (100)	0 (0)	
STK11/TP53						
Not detected	16 (37.2)	27 (62.8)	1	26 (60.5)	17 (39.5)	0.398
Detected	3 (42.9)	4 (57.1)		6 (85.7)	1 (14.3)	

**Table 4:** Associations between PD-L1 expression, TMB, and genetic alterations.

Alteration	PD-L1		*p*	PD-L1		*p*	TMB		*p*
	Negative	Positive		Low	High		Low	High	
EGFR									
WT	18 (38.3)	29 (61.7)	0.29	31 (66)	16 (34)	0.125	28 (63.6)	17 (36.4)	1
MT	1 (100)	0 (0)		1 (100)	0 (0)		1 (100)	0 (0)	
Amplification	0 (0)	2 (100)		0 (0)	2 (100)		1 (50)	1 (50)	
ALK									
WT	18 (37.5)	30 (62.5)	1	31 (64.6)	17 (35.4)	1	28 (62.2)	17 (37.8)	0.528
MT	1 (50)	1 (50)		1 (50)	1 (50)		2 (100)	0 (0)	
BRAF									
WT	18 (37.5)	30 (62.5)	1	31 (64.6)	17 (35.4)	1	29 (63)	17 (37)	0.528
MT	1 (50)	1 (50)		1 (50)	1 (50)		1 (100)	0 (0)	
ERBB2									
WT	16 (36.4)	28 (63.6)	0.605	28 (63.6)	16 (36.4)	1	27 (65.9)	14 (34.1)	0.581
MT	1 (5.3)	0 (0)		1 (102)	0 (0)		1 (100)	0 (0)	
Amplification	2 (40)	3 (60)		3 (60)	2 (40)		2 (40)	3 (60)	
KRAS									
WT	12 (38.7)	19 (61.3)	0.777	18 (58.1)	13 (41.9)	0.373	18 (60)	12 (40)	0.576
MT	7 (41.2)	10 (58.8)		13 (76.5)	4 (23.5)		11 (73.3)	4 (26.7)	
Amplification	0 (0)	2 (100)		1 (50)	1 (50)		1 (50)	1 (50)	
MET									
WT	18 (37.5)	30 (62.5)	0.62	31 (64.6)	17 (35.4)	0.595	29 (64.4)	16 (35.6)	0.598
MT	1 (100)	0 (0)		1 (100)	0 (0)		0 (0)	1 (100)	
Amplification	0 (0)	1 (100)		0 (0)	1 (100)		1 (100)	0 (0)	
RET									
WT	18 (36.7)	31 (63.6)	0.38	31 (63.3)	18 (36.7)	1	29 (63)	17 (37)	1
MT	1 (100)	0 (0)		1 (100)	0 (0)		1 (100)	0 (0)	
ROS1									
WT	19 (36.6)	29 (60.4)	0.519	32 (66.7)	16 (33.3)	0.125	28 (62.2)	17 (37.8)	0.528
MT	0 (0)	2 (100)		0 (0)	2 (100)		2 (100)	0 (0)	
STK11									
WT	12 (30.8)	27 (69.2)	0.078	22 (56.4)	17 (43.6)	0.072	25 (67.6)	12 (32.4)	0.46
MT	7 (63.6)	4 (36.4)		10 (90.9)	1 (9.1)		5 (50)	5 (50)	
TP53									
WT	12 (54.5)	10 (45.5)	0.043	18 (81.8)	4 (18.2)	0.036	16 (80)	4 (20)	0.067
MT	7 (25)	21 (75)		14 (50)	14 (50)		14 (51.9)	13 (48.1)	
ZNF703									
WT	18 (38.3)	29 (61.7)	1	29 (61.7)	18 (38.3)	0.544	30 (68.2)	14 (31.8)	0.042
Amplification	1 (33.3)	2 (66.7)		3 (100)	0 (0)		0 (0)	3 (100)	
TERC									
WT	17 (37)	29 (63)	0.629	29 (63)	17 (37)	1	28 (65.1)	15 (34.9)	0.613
Amplification	2 (50)	2 (50)		3 (75)	1 (25)		2 (50)	2 (50)	
MTAP									
WT	12 (34.3)	23 (65.7)	0.528	21 (60)	14 (40)	0.523	18 (56.3)	14 (43.8)	0.193
MT	7 (46.7)	8 (53.3)		11 (73.3)	4 (26.7)		12 (80)	3 (20)	
CDKN2A/B									
WT	10 (38.5)	16 (61.5)	1	18 (69.2)	8 (30.8)	0.557	12 (52.2)	11 (47.8)	0.135
MT	9 (37.5)	15 (62.5)		14 (58.3)	10 (41.7)		18 (75)	6 (25)	
FGFR1									
WT	18 (39.1)	28 (60.9)	1	30 (65.2)	16 (34.8)	0.612	28 (65.1)	15 (34.9)	0.613
Amplification	1 (25)	3 (75)		2 (50)	2 (50)		2 (50)	2 (50)	
KEAP1									
WT	15 (33.3)	30 (66.7)	0.062	28 (62.2)	17 (37.8)	0.642	29 (69)	13 (31)	0.051
MT	4 (80)	1 (20)		4 (80)	1 (20)		1 (20)	4 (80)	
KMT2D/MLL2									
WT	17 (37)	29 (63)	0.629	30 (65.2)	16 (34.8)	0.612	28 (65.1)	15 (34.9)	0.613
MT	2 (50)	2 (50)		2 (50)	2 (50)		2 (50)	2 (50)	
NSD3/WHSC1L1									
WT	18 (39.1)	28 (60.9)	1	30 (65.2)	16 (34.8)	0.612	28 (65.1)	15 (34.9)	0.613
Amplification	1 (25)	3 (75)		2 (50)	2 (50)		2 (50)	2 (50)	
RBM10									
WT	17 (37.8)	28 (62.2)	1	28 (62.2)	17 (37.8)	0.642	28 (65.1)	15 (34.9)	0.613
MT	2 (40)	3 (60)		4 (80)	1 (20)		2 (50)	2 (50)	
CDK4									
WT	17 (37)	29 (63)	0.629	29 (63)	17 (37)	1	29 (67.4)	14 (32.6)	0.128
Amplification	2 (50)	2 (50)		3 (25)	1 (25)		1 (25)	3 (75)	
MDM2									
WT	15 (33.3)	30 (66.7)	0.062	28 (62.2)	17 (37.8)	0.642	27 (64.3)	15 (35.7)	1
Amplification	4 (80)	1 (20)		4 (80)	1 (20)		3 (60)	2 (40)	
PIK3CA									
WT	16 (35.6)	29 (64.4)	0.355	28 (62.2)	17 (37.8)	0.642	25 (59.5)	17 (40.5)	0.143
MT	3 (60)	2 (40)		4 (80)	1 (20)		5 (100)	0 (0)	
EPHB4									
WT	17 (37)	29 (63)	0.629	28 (60.9)	18 (39.1)	0.283	29 (67.4)	14 (32.6)	0.128
Amplification	2 (50)	2 (50)		4 (100)	0 (0)		1 (25)	3 (75)	
NKX2/1									
WT	18 (39.1)	28 (60.9)	1	29 (63)	17 (37)	1	29 (67.4)	14 (32.6)	0.128
Amplification	1 (25)	3 (75)		3 (75)	1 (25)		1 (25)	3 (75)	
APC									
WT	19 (40.4)	28 (59.6)	0.279	30 (63.8)	17 (36.2)	1	30 (68.2)	14 (31.8)	0.042
MT	0 (0)	3 (100)		2 (66.7)	1 (33.3)		0 (0)	3 (100)	
CEHK2									
WT	19 (40.4)	28 (59.6)	0.279	30 (63.8)	17 (36.2)	1	30 (68.2)	14 (31.8)	0.042
MT	0 (0)	3 (100)		2 (66.7)	1 (33.3)		0 (0)	3 (100)	
MYC									
WT	18 (38.3)	29 (61.7)	1	29 (61.7)	18 (38.3)	0.544	28 (63.6)	16 (36.4)	1
Amplification	1 (33.3)	2 (66.7)		3 (100)	0 (0)		2 (66.7)	1 (33.3)	
CRKL									
WT	17 (36.2)	30 (63.8)	0.549	29 (61.7)	18 (38.3)	0.544	29 (65.9)	15 (34.1)	0.544
Amplification	2 (66.7)	1 (33.3)		3 (100)	0 (0)		1 (33.3)	2 (66.7)	
PRKCI									
WT	17 (36.2)	30 (63.8)	0.549	30 (63.8)	17 (36.2)	1	28 (63.6)	16 (36.4)	1
Amplification	2 (66.7)	1 (33.3)		2 (66.7)	1 (33.3)		2 (66.7)	1 (33.3)	
TET2									
WT	17 (36.2)	30 (63.8)	0.549	29 (61.7)	18 (38.3)	0.544	28 (63.6)	16 (36.4)	1
MT	2 (66.7)	1 (33.3)		3 (100)	0 (0)		2 (66.7)	1 (33.3)	
ARID1A									
WT	18 (38.3)	29 (61.7)	1	31 (66)	16 (34)	0.291	29 (65.9)	15 (34.1)	0.544
MT	1 (33.3)	2 (66.7)		1 (33.3)	2 (66.7)		1 (33.3)	2 (66.7)	
NORCH2									
WT	18 (38.3)	29 (61.7)	1	29 (61.7)	18 (38.3)	0.544	30 (68.2)	14 (31.8)	0.042
MT	1 (33.3)	2 (66.7)		3 (100)	0 (0)		0 (0)	3 (100)	
ATM									
WT	19 (40.4)	28 (59.6)	0.279	30 (63.8)	17 (36.2)	1	29 (64.4)	16 (35.6)	1
MT	0 (0)	3 (100)		2 (66.7)	1 (33.3)		1 (50)	1 (50)	
BRCA1									
WT	18 (38.3)	29 (61.7)	1	31 (66)	16 (34)	0.291	27 (61.4)	17 (38.6)	0.292
MT	1 (33.3)	2 (66.7)		1 (33.3)	2 (66.7)		3 (100)	0 (0)	

PD-L1—programmed death ligand 1, TMB—tumour mutation burden, WT—wild type, MT—mutation.

**Table 5:** Clinicopathological and molecular characteristics of patients with fast disease progression.

Clinical Case	Histological Type	PD-L1 Expression (%)	TMB (muts/Mb)	Treatment	STK11 MT	KEAP1 MT	Other Alterations
I	Adenocarcinoma	60	10	pembrolizumab	+	−	TP53 MT, CDKN2A/B MT
II	Squamous cell carcinoma	80	6	pembrolizumab	−	−	TP53 MT, KRAS amplifications, FGFR1 amplifications, NSD3/WHSC1L1 amplifications, KMT2D/MLL2 MT
III	Adenocarcinoma	60	17	Carboplatin, pemetrexed, pembrolizumab	−	+	TP53 MT CDKN2A/B MT, APC MT
IV	Adenocarcinoma	0	10	Carboplatin, pemetrexed, pembrolizumab	−	+	TERC MT EPHB4 MT NKX2/1 MT CRKL MT PRKCI MT NOTCH MT
V	Adenocarcinoma	0	6	Carboplatin, pemetrexed, pembrolizumab	−	−	ERBB2 amplification TP53 MT CDKN2B MT TET2 MT BRCA1 MT
VI	Large cell carcinoma	0	22	Carboplatin, pemetrexed, pembrolizumab	+	+	ERBB2 amplification TP53 MT CDK4 amplification EPHB4 MT
VII	Adenocarcinoma	90	4	Pembrolizumab	−	−	ERBB2 MT ERBB2 amplification TP53 MT MTAP MT CDKN2A/B MT
VIII	Adenocarcinoma	2	13	Carboplatin, pemetrexed, pembrolizumab	+	−	KRAS amplification TP53 MT

PD-L1—programmed cell death ligand 1, TMB—tumour mutation burden, MT—mutation, “+”—mutation was found, “−“—mutation was not found.

**Table 6:** Differences between the non-fast progression and fast progression subgroups.

	Non–Fast Progression	Fast Progression	OR	95% CI	*p*
n	28	8			
Sex					
Male	20 (71.4)	8 (28.6)	0.714	0.565–0.903	0.156
Female	8 (100)	0 (0)			
Age group					
<65 years	12 (85.7)	2 (14.3)	2.25	0.385–13.166	0.441
≥65 years	16 (72.7)	6 (27.3)			
Smoking status					
Non-smokers	4 (80)	1 (20)	1.167	0.112–12.202	1
Current or former smokers	24 (77.4)	7 (22.6)			
COPD					
Absent	20 (74.1)	7 (25.9)	0.357	0.038–3.388	0.648
Present	8 (88.9)	1 (11.1)			
Histological NSCLC type					
Adenocarcinoma	18 (75)	6 (25)	0.333	0.035–3.205	0.644
Squamous cell carcinoma	9 (90)	1 (10)			
Differentiation					
Well-moderate	14 (87.5)	2 (12.5)	4.375	0.684–27.983	0.192
Poor-undifferentiated	8 (61.5)	5 (38.5)			
NSCLC stage					
IVa	14 (82.4)	3 (17.6)	1.667	0.333–8.352	0.695
IVb	14 (73.7)	5 (26.3)			
T status					
T1-3	14 (73.7)	5 (26.3)	0.6	0.12–3.007	0.695
T4	14 (82.4)	3 (17.6)			
Lymph node status					
N0	3 (100)	0 (0)	1.32	1.088–1.601	1
N1-3	25 (75.8)	8 (24.2)			
Metastases					
M1a	10 (83.3)	2 (16.7)	1.667	0.282–9.856	0.691
M1b-1c	18 (75)	6 (25)			
TMB					
low	17 (85)	3 (15)	3.148	0.608–16.289	0.228
high	9 (64.3)	5 (35.7)			
PD-L1					
negative	11 (78.6)	3 (21.4)	1.078	0.213–0.713	1
positive	17 (77.3)	5 (22.7)			
STK11					
WT	22 (81.5)	5 (18.5)	2.2	0.405–11.949	0.384
MT	6 (66.7)	3 (33.3)			
KEAP1					
WT	27 (84.4)	5 (15.6)	16.2	1.38–188.883	0.028
MT	1 (25)	3 (75)			
MDM2					
WT	26 (76.5)	8 (23.5)	0.765	0.635–0.921	1
amplification	2 (100)	0 (0)			
ERBB2					
WT	25 (83.3)	5 (16.7)	7.5	0.984–57.138	0.067
amplification	2 (40)	3 (60)			
TP53					
WT	11 (91.7)	1 (8.3)	4.529	0.488–42.052	0.224
MT	17 (70.8)	7 (29.2)			

COPD—chronic obstructive pulmonary disease, NSCLC—non-small cell lung cancer, PD-L1—programmed death ligand 1, TMB—tumour mutation burden, MT—mutation, WT—wild type, OR—odds ratio, CI—confidence interval.

## Data Availability

The data presented in this study are available upon request from the corresponding author.

## Supplementary Materials

The following supporting information can be downloaded at: https://www.mdpi.com/article/10.3390/ijms26136348/s1.

## References

[1] Lee S.M., Schulz C., Prabhash K., Kowalski D., Szczesna A., Han B., Rittmeyer A., Talbot T., Vicente D., Califano R. (2023). First-line atezolizumab monotherapy versus single-agent chemotherapy in patients with non-small-cell lung cancer ineligible for treatment with a platinum-containing regimen (IPSOS): A phase 3, global, multicentre, open-label, randomised controlled study. Lancet.

[2] Belaroussi Y., Bouteiller F., Bellera C., Pasquier D., Perol M., Debieuvre D., Filleron T., Girard N., Schott R., Mathoulin-Pélissier S. (2023). Survival outcomes of patients with metastatic non-small cell lung cancer receiving chemotherapy or immunotherapy as first-line in a real-life setting. Sci. Rep..

[3] Spigel D., De Marinis F., Giaccone G., Reinmuth N., Vergnenegre A., Barrios C.H., Morise M., Felip E., Andric Z.G., Geater S. (2019). IMpower110: Interim overall survival (OS) analysis of a phase III study of atezolizumab (atezo) vs platinum-based chemotherapy (chemo) as first-line (1L) treatment (tx) in PD-L1–selected NSCLC. Ann. Oncol..

[4] Felip E., Altorki N., Zhou C., Vallières E., Martínez-Martí A., Rittmeyer A., Chella A., Reck M., Goloborodko O., Huang M. (2023). Overall survival with adjuvant atezolizumab after chemotherapy in resected stage II-IIIA non-small-cell lung cancer (IMpower010): A randomised, multicentre, open-label, phase III trial. Ann. Oncol..

[5] O’Brien M., Paz-Ares L., Marreaud S., Dafni U., Oselin K., Havel L., Esteban E., Isla D., Martinez-Marti A., Faehling M. (2022). Pembrolizumab versus placebo as adjuvant therapy for completely resected stage IB–IIIA non-small-cell lung cancer (PEARLS/KEYNOTE-091): An interim analysis of a randomised, triple-blind, phase 3 trial. Lancet Oncol..

[6] Wakelee H., Liberman M., Kato T., Tsuboi M., Lee S.-H., Gao S., Chen K.-N., Dooms C., Majem M., Eigendorff E. (2023). Perioperative Pembrolizumab for Early-Stage Non–Small-Cell Lung Cancer. N. Engl. J. Med..

[7] Kanwal B., Biswas S., Seminara R.S., Jeet C. (2018). Immunotherapy in Advanced Non-small Cell Lung Cancer Patients: Ushering Chemotherapy Through the Checkpoint Inhibitors?. Cureus.

[8] Putzu C., Canova S., Paliogiannis P., Lobrano R., Sala L., Cortinovis D.L., Colonese F. (2023). Duration of Immunotherapy in Non-Small Cell Lung Cancer Survivors: A Lifelong Commitment?. Cancers.

[9] Garassino M.C., Gadgeel S., Speranza G., Felip E., Esteban E., Dómine M., Hochmair M.J., Powell S.F., Bischoff H.G., Peled N. (2023). Pembrolizumab Plus Pemetrexed and Platinum in Nonsquamous Non–Small-Cell Lung Cancer: 5-Year Outcomes From the Phase 3 KEYNOTE-189 Study. J. Clin. Oncol..

[10] Incorvaia L., Fanale D., Badalamenti G., Barraco N., Bono M., Corsini L.R., Galvano A., Gristina V., Listì A., Vieni S. (2019). Programmed Death Ligand 1 (PD-L1) as a Predictive Biomarker for Pembrolizumab Therapy in Patients with Advanced Non-Small-Cell Lung Cancer (NSCLC). Adv. Ther..

[11] Catalano M., Iannone L.F., Nesi G., Nobili S., Mini E., Roviello G. (2023). Immunotherapy-related biomarkers: Confirmations and uncertainties. Crit. Rev. Oncol. Hematol..

[12] Vryza P., Fischer T., Mistakidi E., Zaravinos A. (2023). Tumor mutation burden in the prognosis and response of lung cancer patients to immune-checkpoint inhibition therapies. Transl. Oncol..

[13] Di Federico A., De Giglio A., Gelsomino F., Sperandi F., Melotti B., Ardizzoni A. (2023). Predictors of survival to immunotherapy and chemoimmunotherapy in non-small cell lung cancer: A meta-analysis. JNCI: J. Natl. Cancer Inst..

[14] Alessi J.V., Elkrief A., Ricciuti B., Wang X., Cortellini A., Vaz V.R., Lamberti G., Frias R.L., Venkatraman D., Fulgenzi C.A.M. (2023). Clinicopathologic and Genomic Factors Impacting Efficacy of First-Line Chemoimmunotherapy in Advanced NSCLC. J. Thorac. Oncol..

[15] National Comprehensive Cancer Network. NCCN Clinical Practice Guidelines in Oncology: Lung Cancer—Metastatic, Patient Version [Internet]. Fort Washington (PA): NCCN; 2024. https://www.nccn.org/patients/guidelines/content/PDF/lung-metastatic-patient.pdf.

[16] Saller J.J., Boyle T.A. (2022). Molecular Pathology of Lung Cancer. Cold Spring Harb. Perspect. Med..

[17] De Giglio A., De Biase D., Favorito V., Maloberti T., Di Federico A., Zacchini F., Venturi G., Parisi C., Gustavo Dall’Olio F., Ricciotti I. (2025). STK11 mutations correlate with poor prognosis for advanced NSCLC treated with first-line immunotherapy or chemo-immunotherapy according to KRAS, TP53, KEAP1, and SMARCA4 status. Lung Cancer.

[18] Shire N.J., Klein A.B., Golozar A., Collins J.M., Fraeman K.H., Nordstrom B.L., McEwen R., Hembrough T., Rizvi N.A. (2020). STK11 (LKB1) mutations in metastatic NSCLC: Prognostic value in the real world. PLoS ONE.

[19] Ricciuti B., Arbour K.C., Lin J.J., Vajdi A., Vokes N., Hong L., Zhang J., Tolstorukov M.Y., Li Y.Y., Spurr L.F. (2022). Diminished Efficacy of Programmed Death-(Ligand)1 Inhibition in STK11- and KEAP1-Mutant Lung Adenocarcinoma Is Affected by KRAS Mutation Status. J. Thorac. Oncol..

[20] Sun L., Handorf E.A., Zhou Y., Borghaei H., Aggarwal C., Bauman J. (2024). Outcomes in patients treated with frontline immune checkpoint inhibition (ICI) for advanced NSCLC with KRAS mutations and STK11/KEAP1 comutations across PD-L1 levels. Lung Cancer.

[21] Van De Haar J., Mankor J.M., Hummelink K., Monkhorst K., Smit E.F., Wessels L.F.A., Cuppen E., Aerts J.G.J.V., Voest E.E. (2024). Combining Genomic Biomarkers to Guide Immunotherapy in Non–Small Cell Lung Cancer. Clin. Cancer Res..

[22] Gandara D., Reck M., Moro-Sibilot D., Mazieres J., Gadgeel S., Morris S., Cardona A., Mendus D., Ballinger M., Rittmeyer A. (2021). Fast progression in non–small cell lung cancer: Results from the randomized phase III OAK study evaluating second-line atezolizumab versus docetaxel. J. Immunother. Cancer.

[23] Ferrara R., Mezquita L., Texier M., Lahmar J., Audigier-Valette C., Tessonnier L., Mazieres J., Zalcman G., Brosseau S., Le Moulec S. (2020). Comparison of Fast-Progression, Hyperprogressive Disease, and Early Deaths in Advanced Non–Small-Cell Lung Cancer Treated with PD-1/PD-L1 Inhibitors or Chemotherapy. JCO Precis. Oncol..

[24] Passaro A., Novello S., Giannarelli D., Bria E., Galetta D., Gelibter A., Reale M.L., Carnio S., Vita E., Stefani A. (2021). Early Progression in Non-Small Cell Lung Cancer (NSCLC) with High PD-L1 Treated with Pembrolizumab in First-Line Setting: A Prognostic Scoring System Based on Clinical Features. Cancers.

[25] Herbst R.S., Garon E.B., Kim D.-W., Cho B.C., Gervais R., Perez-Gracia J.L., Han J.-Y., Majem M., Forster M.D., Monnet I. (2021). Five Year Survival Update From KEYNOTE-010: Pembrolizumab Versus Docetaxel for Previously Treated, Programmed Death-Ligand 1–Positive Advanced NSCLC. J. Thorac. Oncol..

[26] Hendriks L.E., Rouleau E., Besse B. (2018). Clinical utility of tumor mutational burden in patients with non-small cell lung cancer treated with immunotherapy. Transl. Lung Cancer Res..

[27] Willis C., Bauer H., Au T.H., Menon J., Unni S., Tran D., Rivers Z., Akerley W., Schabath M.B., Badin F. (2022). Real-world survival analysis by tumor mutational burden in non-small cell lung cancer: A multisite U.S. study. Oncotarget.

[28] Sha D., Jin Z., Budczies J., Kluck K., Stenzinger A., Sinicrope F.A. (2020). Tumor Mutational Burden as a Predictive Biomarker in Solid Tumors. Cancer Discov..

[29] Remon J., Collazo A., Jimenez B. (2020). CheckMate 227 trial has not checked the immune-strategy in first-line setting in advanced non-small cell lung cancer. Transl. Cancer Res..

[30] Cascetta P., Marinello A., Lazzari C., Gregorc V., Planchard D., Bianco R., Normanno N., Morabito A. (2022). KRAS in NSCLC: State of the Art and Future Perspectives. Cancers.

[31] Canale M., Andrikou K., Priano I., Cravero P., Pasini L., Urbini M., Delmonte A., Crinò L., Bronte G., Ulivi P. (2022). The Role of TP53 Mutations in EGFR-Mutated Non-Small-Cell Lung Cancer: Clinical Significance and Implications for Therapy. Cancers.

[32] Tseng Y.-H., Tran T.T.M., Tsai Chang J., Huang Y.-T., Nguyen A.T., Chang I.Y., Chen Y.-T., Hsieh H.-W., Juang Y.-L., Chang P.M.-H. (2025). Utilizing TP53 hotspot mutations as effective predictors of gemcitabine treatment outcome in non-small-cell lung cancer. Cell Death Discov..

[33] Di Federico A., De Giglio A., Parisi C., Gelsomino F. (2021). STK11/LKB1 and KEAP1 mutations in non-small cell lung cancer: Prognostic rather than predictive?. Eur. J. Cancer.

[34] Brune M.M., Savic Prince S., Vlajnic T., Chijioke O., Roma L., König D., Bubendorf L. (2024). MTAP as an emerging biomarker in thoracic malignancies. Lung Cancer.

[35] Peng Y., Chen Y., Song M., Zhang X., Li P., Yu X., Huang Y., Zhang N., Ji L., Xia L. (2022). Co-occurrence of CDKN2A/B and IFN-I homozygous deletions correlates with an immunosuppressive phenotype and poor prognosis in lung adenocarcinoma. Mol. Oncol..

[36] Best S.A., Gubser P.M., Sethumadhavan S., Kersbergen A., Negrón Abril Y.L., Goldford J., Sellers K., Abeysekera W., Garnham A.L., McDonald J.A. (2022). Glutaminase inhibition impairs CD8 T cell activation in STK11-/Lkb1-deficient lung cancer. Cell Metab..

[37] Papalexi E., Mimitou E.P., Butler A.W., Foster S., Bracken B., Mauck W.M., Wessels H.-H., Hao Y., Yeung B.Z., Smibert P. (2021). Characterizing the molecular regulation of inhibitory immune checkpoints with multimodal single-cell screens. Nat. Genet..

[38] Long Y., Yu X., Chen R., Tong Y., Gong L. (2022). Noncanonical PD-1/PD-L1 Axis in Relation to the Efficacy of Anti-PD Therapy. Front. Immunol..

[39] Ricciuti B., Garassino M.C. (2024). Precision Immunotherapy for STK11/KEAP1-Mutant NSCLC. J. Thorac. Oncol..

[40] Jeong Y., Hellyer J.A., Stehr H., Hoang N.T., Niu X., Das M., Padda S.K., Ramchandran K., Neal J.W., Wakelee H. (2020). Role of KEAP1/NFE2L2 Mutations in the Chemotherapeutic Response of Patients with Non–Small Cell Lung Cancer. Clin. Cancer Res..

[41] Lamberti G., Spurr L.F., Li Y., Ricciuti B., Recondo G., Umeton R., Nishino M., Sholl L.M., Meyerson M.L., Cherniack A.D. (2020). Clinicopathological and genomic correlates of programmed cell death ligand 1 (PD-L1) expression in nonsquamous non-small-cell lung cancer. Ann. Oncol..

[42] Garassino M.C., Gadgeel S., Novello S., Halmos B., Felip E., Speranza G., Hui R., Garon E.B., Horinouchi H., Sugawara S. (2023). Associations of Tissue Tumor Mutational Burden and Mutational Status With Clinical Outcomes With Pembrolizumab Plus Chemotherapy Versus Chemotherapy For Metastatic NSCLC. JTO Clin. Res. Rep..

[43] Skoulidis F., Goldberg M.E., Greenawalt D.M., Hellmann M.D., Awad M.M., Gainor J.F., Schrock A.B., Hartmaier R.J., Trabucco S.E., Gay L. (2018). *STK11/LKB1* Mutations and PD-1 Inhibitor Resistance in *KRAS* -Mutant Lung Adenocarcinoma. Cancer Discov..

[44] Biton J., Mansuet-Lupo A., Pécuchet N., Alifano M., Ouakrim H., Arrondeau J., Boudou-Rouquette P., Goldwasser F., Leroy K., Goc J. (2018). *TP53*, *STK11*, and *EGFR* Mutations Predict Tumor Immune Profile and the Response to Anti–PD-1 in Lung Adenocarcinoma. Clin. Cancer Res..

[45] Lamberti G., Sisi M., Andrini E., Palladini A., Giunchi F., Lollini P.-L., Ardizzoni A., Gelsomino F. (2020). The Mechanisms of PD-L1 Regulation in Non-Small-Cell Lung Cancer (NSCLC): Which Are the Involved Players?. Cancers.

[46] Tabernero J., Hyman D.M., Soria J.-C. (2021). ULK1 Inhibition Restores Antigen Presentation in *LKB1* -Mutant Lung Cancer. Cancer Discov..

[47] Pons-Tostivint E., Lugat A., Fontenau J.-F., Denis M.G., Bennouna J. (2021). STK11/LKB1 Modulation of the Immune Response in Lung Cancer: From Biology to Therapeutic Impact. Cells.

[48] Shackelford D.B., Shaw R.J. (2009). The LKB1–AMPK pathway: Metabolism and growth control in tumour suppression. Nat. Rev. Cancer.

[49] Frille A., Boeschen M., Wirtz H., Stiller M., Bläker H., Von Laffert M. (2024). TP53 co-mutations in advanced lung adenocarcinoma: Comparative bioinformatic analyses suggest ambivalent character on overall survival alongside KRAS, STK11 and KEAP1 mutations. Front. Oncol..

[50] Sun H., Liu S.-Y., Zhou J.-Y., Xu J.-T., Zhang H.-K., Yan H.-H., Huan J.-J., Dai P.-P., Xu C.-R., Su J. (2020). Specific TP53 subtype as biomarker for immune checkpoint inhibitors in lung adenocarcinoma. EBioMedicine.

[51] Ma K., Huang F., Wang Y., Kang Y., Wang Q., Tang J., Sun P., Lou J., Qiao R., Si J. (2022). Relationship between tumor mutational burden, gene mutation status, and clinical characteristics in 340 cases of lung adenocarcinoma. Cancer Med..

[52] Fu J., Li Y., Li C., Tong Y., Li M., Cang S. (2021). A special prognostic indicator: Tumor mutation burden combined with immune infiltrates in lung adenocarcinoma with TP53 mutation. Transl. Cancer. Res..

[53] Cortez M.A., Ivan C., Valdecanas D., Wang X., Peltier H.J., Ye Y., Araujo L., Carbone D.P., Shilo K., Giri D.K. (2016). PDL1 Regulation by p53 via miR-34. JNCI J. Natl. Cancer Inst..

[54] Martinkova L., Zatloukalova P., Kucerikova M., Friedlova N., Tylichova Z., Zavadil-Kokas F., Hupp T.R., Coates P.J., Vojtesek B. (2024). Inverse correlation between TP53 gene status and PD-L1 protein levels in a melanoma cell model depends on an IRF1/SOX10 regulatory axis. Cell Mol. Biol. Lett..

[55] Hong L., Patel S., Drusbosky L.M., Xiong Y., Chen R., Geng R., Heeke S., Nilsson M., Wu J., Heymach J.V. (2024). Molecular landscape of ERBB2 alterations in 3000 advanced NSCLC patients. NPJ Precis. Oncol..

[56] Shih J.-Y. (2024). ERBB2 Amplification in NSCLC: How Many Faces?. J. Thorac. Oncol..

[57] Zhao S., Xian X., Tian P., Li W., Wang K., Li Y. (2021). Efficacy of Combination Chemo-Immunotherapy as a First-Line Treatment for Advanced Non-Small-Cell Lung Cancer Patients With HER2 Alterations: A Case Series. Front. Oncol..

[58] Bertero L., Massa F., Metovic J., Zanetti R., Castellano I., Ricardi U., Papotti M., Cassoni P. (2018). Eighth Edition of the UICC Classification of Malignant Tumours: An overview of the changes in the pathological TNM classification criteria—What has changed and why?. Virchows Arch..

[59] Nicholson A.G., Tsao M.S., Beasley M.B., Borczuk A.C., Brambilla E., Cooper W.A., Dacic S., Jain D., Kerr K.M., Lantuejoul S. (2022). The 2021 WHO Classification of Lung Tumors: Impact of Advances Since 2015. J. Thorac. Oncol..

[60] Marcus L., Fashoyin-Aje L.A., Donoghue M., Yuan M., Rodriguez L., Gallagher P.S., Philip R., Ghosh S., Theoret M.R., Beaver J.A. (2021). FDA Approval Summary: Pembrolizumab for the Treatment of Tumor Mutational Burden–High Solid Tumors. Clin. Cancer Res..

[61] Eisenhauer E.A., Therasse P., Bogaerts J., Schwartz L.H., Sargent D., Ford R., Dancey J., Arbuck S., Gwyther S., Mooney M. (2009). New response evaluation criteria in solid tumours: Revised RECIST guideline (version 1.1). Eur. J. Cancer.

