# Supplementary material for: PD-L1 Expression and Comprehensive Genomic Profiling in Advanced NSCLC: A Single-Centre Experience
Source: Int J Mol Sci. 2025 Jul 1;26(13):6348. doi: 10.3390/ijms26136348 (PMC12249841; doi:10.3390/ijms26136348)
Supplement: Supplementary file 1 [file ijms-26-06348-s001.zip › ijms-3674493-supplementary.pdf]

Figure S1. TMB presented in percentages.

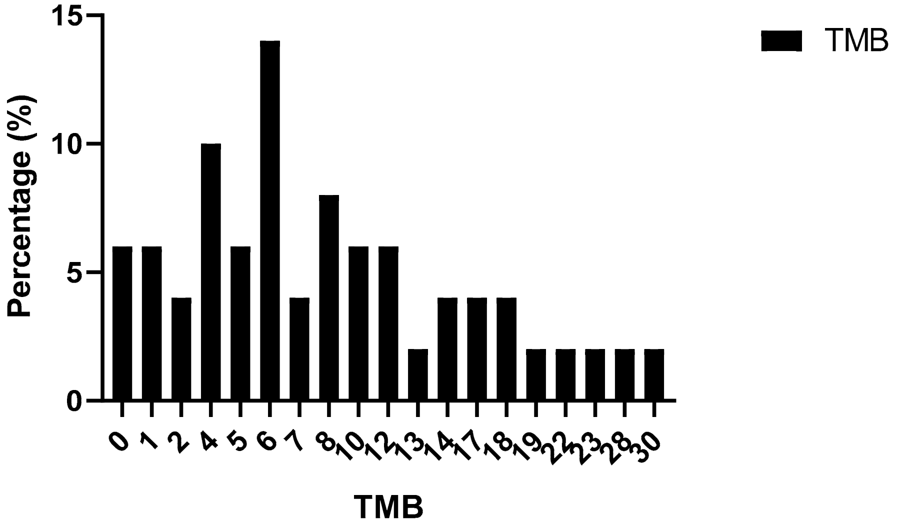

TMB – tumour mutation burden.

Figure S2. Association between high or low TMB level and packyears.

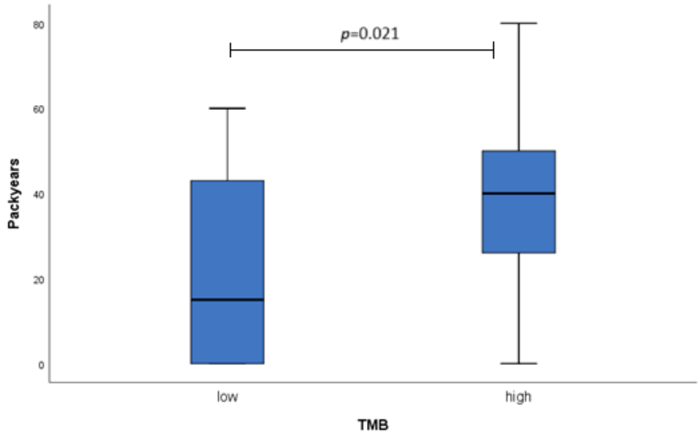

TMB – tumour mutation burden.

Table S1. Low or high TMB level and clinicopathological characteristics.

	TMB		
	low	high	*p*
Age, n (%)			
<65 years	17 (81)	4 (19)	0.004
≥65 years	13 (50)	13 (50)	
Gender, n (%)			
women	11 (100)	0 (0)	0.036
men	19 (52.8)	17 (47.2)	

Smoking status, n (%)			
Non-smokers	9 (90)	1 (10)	0.07
Smokers	21 (56.8)	16 (43.2)	
COPD, n (%)			
absent	26 (70.3)	11 (29.7)	0.136
present	4 (40)	6 (60)	
Histological NSCLC type, n (%)			
adenocarcinoma	23 (65.7)	12 (34.3)	0.726
Squamous cell carcinoma	6 (60)	4 (40)	
Differentiation, n (%)			
Well-moderate	15 (75)	5 (25)	0.188
Poor-undifferentiated	9 (52.9)	8 (47.1)	
NSCLC stage, n (%)			
IVa	12 (60)	8 (40)	0.638
IVb	18 (66.7)	9 (33.3)	
Tumour size			
T1-3	13 (54.2)	11 (45.8)	0.159
T4	17 (73.9)	6 (26.1)	
Lymph node status, n (%)			
N0-2	13 (61.9)	8 (38.1)	0.805
N3	17 (65.4)	9 (34.6)	
Metastases			
M1a-1b	12 (60)	8 (40)	0.638
M1c	18 (66.7)	9 (33.3)	

TMB – tumour mutation burden, COPD – chronic obstructive pulmonary disease, NSCLC – non small cell lung cancer.

Table S2. Comparison of radiological response and PD-L1 alone or together with most frequent mutations.

	PR	SD	PD	*p*
PD-L1, n (%)				
Negative	1 (7.1)	9 (64.3)	4 (28.6)	0.082
Positive	9 (40.9)	8 (36.4)	5 (22.7)	
PD-L1$^-$/KRAS^{WT}	1 (11.1)	4 (44.4)	4 (44.4)	0.167
PD-L1$^-$/KRAS^{MT}	0 (0)	5 (100)	0 (0)	
PD-L1$^+$/KRAS^{WT}	6 (40)	6 (40)	3 (20)	
PD-L1$^+$/KRAS^{MT}	3 (42.9)	2 (28.6)	2 (28.6)	
PD-L1$^-$/STK11^{WT}	1 (12.5)	5 (62.5)	2 (25)	0.426
PD-L1$^-$/STK11^{MT}	0 (0)	3 (60)	2 (40)	
PD-L1$^+$/STK11^{WT}	8 (44.4)	7 (38.9)	3 (16.7)	
PD-L1$^+$/STK11^{MT}	1 (20)	2 (40)	2 (40)	
PD-L1$^-$/CDKN2 A/B^{WT}	1 (12.5)	5 (62.5)	2 (25)	0.508
PD-L1$^-$/CDKN2 A/B^{MT}	0 (0)	4 (66.7)	2 (33.3)	
PD-L1$^+$/CDKN2 A/B ^{WT}	4 (40)	4 (40)	2 (20)	
PD-L1$^+$/CDKN2 A/B^{MT}	5 (41.7)	4 (33.3)	3 (25)	

PD-L1^{-}/MTAP^{WT}	1 (10)	6 (60)	3 (30)	0.343
PD-L1^{-}/MTAP^{MT}	0 (0)	3 (75)	1 (25)	
PD-L1^{+}/MTAP^{WT}	8 (47.1)	5 (29.4)	4 (23.5)	
PD-L1^{+}/MTAP^{MT}	1 (20)	3 (60)	1 (20)	
PD-L1^{-}/TP53^{WT}	0 (0)	7 (77.8)	2 (22.2)	0.168
PD-L1^{-}/TP53^{MT}	1 (20)	2 (40)	2 (40)	
PD-L1^{+}/TP53^{WT}	2 (66.7)	1 (33.3)	0 (0)	
PD-L1^{+}/TP53^{MT}	7 (36.8)	7 (36.8)	5 (26.3)	
PD-L1^{low}/KRAS^{WT}	4 (25)	7 (43.8)	5 (31.3)	0.389
PD-L1^{low}/KRAS^{MT}	2 (25)	6 (75)	0 (0)	
PD-L1^{high}/KRAS^{WT}	3 (30)	3 (30)	4 (40)	
PD-L1^{high}/KRAS^{MT}	1 (50)	1 (50)	0 (0)	
PD-L1^{low}/STK11^{WT}	5 (31.3)	9 (56.3)	2 (12.5)	0.599
PD-L1^{low}/STK11^{MT}	-	-	-	
PD-L1^{high}/STK11^{WT}	-	-	-	
PD-L1^{high}/STK11^{MT}	4 (36.4)	4 (36.4)	3 (27.3)	
PD-L1^{low}/CDKN2 A/B^{WT}	4 (30.8)	6 (46.2)	3 (23.1)	0.591
PD-L1^{low}/CDKN2 A/B^{MT}	2 (18.2)	7 (63.6)	2 (18.2)	
PD-L1^{high}/CDKN2 A/B^{WT}	1 (20)	3 (60)	1 (20)	
PD-L1^{high}/CDKN2 A/B^{MT}	3 (42.9)	1 (14.3)	3 (42.9)	
PD-L1^{low}/MTAP^{WT}	5 (31.3)	7 (43.8)	4 (25)	0.588
PD-L1^{low}/MTAP^{MT}	-	-	-	
PD-L1^{high}/MTAP^{WT}	-	-	-	
PD-L1^{high}/MTAP^{MT}	0 (0)	0 (0)	1 (100)	
PD-L1^{low}/TP53^{WT}	4 (44.4)	5 (55.6)	0 (0)	0.399
PD-L1^{low}/TP53^{MT}	2 (25)	4 (50)	2 (25)	
PD-L1^{high}/TP53^{WT}	1 (50)	0 (0)	1 (50)	
PD-L1^{high}/TP53^{MT}	1 (16.7)	4 (66.7)	1 (16.)	

PR – partial response, SD – stable disease, PD – progressive disease, WT – wild-type, MT – mutation, PD-L1 – programmed death ligand 1.

Table S3. Comparison between radiological response and co-occurring mutations.

	PR	PD	OR	95% CI	*p*
Co-occurring mutations, n (%)					
KRAS/STK11					
not detected	10 (52.6)	9 (47.4)	-	-	-
detected	0 (0)	0 (0)			
KRAS/TP53					
not detected	9 (50)	9 (50)	0.5	0.315-0.794	1
detected	1 (100)	0 (0)			
STK11/KEAP1					
not detected	10 (55.6)	8 (44.4)	0.444	0.265-0.745	0.474
detected	0 (0)	1 (100)			

TP53/STK11					
not detected	9 (60)	6 (40)	4.5	0.374-54.155	0.303
detected	1 (25)	3 (75)			
TP53/CDKN2A/B					
not detected	6 (54.5)	5 (45.5)	1.2	0.194-7.441	1
detected	4 (50)	4 (50)			
MTAP/CDKN2A/B					
not detected	9 (56.3)	7 (48.8)	2.571	0.192-34.473	0.582
detected	1 (33.3)	2 (66.7)			

PR- partial response, SD – stable disease, PD – progressive disease, WT – wild type, MT – mutation.
